# Pediatric Ventilator-Associated Events Before and After a Multicenter Quality Improvement Initiative

**DOI:** 10.1001/jamanetworkopen.2023.46545

**Published:** 2023-12-07

**Authors:** Andrew G. Wu, Gowri Madhavan, Kathy Deakins, Dana Evans, Angela Hayward, Caitlin Pugh, Angela Carter Stutts, Laurie Mustin, Katherine C. Staubach, Patricia Sisson, Maitreya Coffey, Anne Lyren, Grace M. Lee, Sameer Gupta, Lucy Pereira-Argenziano, Gregory P. Priebe

**Affiliations:** 1 Division of Critical Care Medicine, Department of Anesthesiology, Critical Care and Pain Medicine, Boston Children’s Hospital, Boston, Massachusetts; 2 Harvard Medical School, Boston, Massachusetts; 3 Center for Pediatric and Maternal Value, Stanford Medicine Children’s Health, Palo Alto, California; 4 Pediatric Respiratory Care, University Hospitals (UH) Rainbow Babies and Children’s Hospital, Cleveland, Ohio; 5 Respiratory Care, Ann and Robert H. Lurie Children’s Hospital of Chicago, Chicago, Illinois; 6 Now with Advocate Aurora Health, Downers Grove, Illinois; 7 Infection Prevention Control, University of Wisconsin Hospital and Clinics, Madison; 8 Nursing Quality, Monroe Carell Jr Vanderbilt Children’s Hospital, Nashville, Tennessee; 9 Now with Children’s Healthcare of Atlanta, Atlanta, Georgia; 10 Department of Critical Care, Texas Children’s Hospital, Houston; 11 Cincinnati Children’s Hospital Medical Center, Cincinnati, Ohio; 12 James M. Anderson Center for Health Systems Excellence, Cincinnati Children’s Hospital Medical Center, Cincinnati, Ohio; 13 Division of Paediatric Medicine, The Hospital for Sick Children, Toronto, Ontario, Canada; 14 Case Western Reserve University School of Medicine, UH Rainbow Babies and Children’s Hospital, Cleveland, Ohio; 15 Department of Pediatrics, Infectious Disease, Stanford Medicine Children’s Health, Palo Alto, California; 16 Division of Pediatric Critical Care Medicine, Department of Pediatrics, M Health Fairview Masonic Children’s Hospital, Minneapolis, Minnesota; 17 Neonatal Division, Hackensack University Medical Center, New Hyde Park, New York; 18 Division of Infectious Diseases, Department of Pediatrics, Boston Children’s Hospital, Boston, Massachusetts

## Abstract

**Importance:**

Pediatric ventilator-associated events (PedVAEs, defined as a sustained worsening in oxygenation after a baseline period of stability or improvement) are useful for surveillance of complications from mechanical ventilation. It is unclear whether interventions to mitigate known risk factors can reduce PedVAE rates.

**Objective:**

To assess whether adherence to 1 or more test factors in a quality improvement bundle was associated with a reduction in PedVAE rates.

**Design, Setting, and Participants:**

This multicenter quality improvement study obtained data from 2017 to 2020 for patients who were mechanically ventilated and cared for in neonatal, pediatric, and cardiac intensive care units (ICUs). These ICUs were located in 95 hospitals participating in the Children’s Hospitals’ Solutions for Patient Safety (SPS) network in North America. Data analyses were performed between September 2021 and April 2023.

**Intervention:**

A quality improvement bundle consisted of 3 test factors: multidisciplinary apparent cause analysis, daily discussion of extubation readiness, and daily discussion of fluid balance goals. This bundle was distributed to a subgroup of hospitals that volunteered to participate in a collaborative PedVAE prevention initiative under the SPS network guidance in July 2018.

**Main Outcomes and Measures:**

Each SPS network hospital submitted monthly PedVAE rates from January 1, 2017, to May 31, 2020, and test factor data were submitted from July 1, 2018, to May 31, 2020. Analyses focused on hospitals that reliably submitted PedVAE rate data, defined as outcomes data submission through May 31, 2020, for at least 80% of the baseline and postbaseline periods.

**Results:**

Of the 95 hospitals in the SPS network that reported PedVAE data, 21 were grouped in the Pioneer cohort and 74 in the non-Pioneer cohort. Only 12 hospitals (57%) from the 21 Pioneer hospitals and 33 (45%) from the 74 non-Pioneer hospitals were considered to be reliable reporters of outcome data. Among the 12 hospitals, the PedVAE rate decreased from 1.9 to 1.4 events per 1000 ventilator days (absolute rate difference, −0.6; 95% CI, −0.5 to −0.7; *P* < .001). No significant change in the PedVAE rate was seen among the 33 hospitals that reliably submitted PedVAE rates but did not implement the bundle. Of the 12 hospitals, 3 that reliably performed daily discussion of extubation readiness had a decrease in PedVAE rate from 2.6 to 1.2 events per 1000 ventilator days (absolute rate difference, −1.4; 95% CI, −1.0 to −1.7; *P* < .001), whereas the other 9 hospitals that did not implement this discussion did not have a decrease.

**Conclusions and Relevance:**

This study found that a multicenter quality improvement intervention targeting PedVAE risk factors was associated with a substantial reduction in the rate of PedVAEs in hospital ICUs. The findings suggest that ICU teams seeking to reduce PedVAEs incorporate daily discussion of extubation readiness during morning rounds.

## Key Points

**Question:**

Is a quality improvement bundle that targets identified risk factors for pediatric ventilator-associated events (PedVAEs) associated with reduced rate of these events?

**Findings:**

In this quality improvement study of 95 hospitals with more than 1.4 million ventilator days, a quality improvement bundle decreased the rate of PedVAEs by 26% in hospitals that received training and education on PedVAE prevention compared with hospitals that did not implement such interventions.

**Meaning:**

Findings of this study suggest that improving patient safety through prevention of PedVAEs in hospital intensive care units is possible with implementation of bundled interventions targeting PedVAE risk factors.

## Teaser

This quality improvement study reports the outcome associated with implementing a multidisciplinary apparent cause analysis, daily discussion of extubation readiness, and daily discussion of fluid balance goals in hospital intensive care units.

## Introduction

Hundreds of thousands of children in the US receive mechanical ventilation annually^1,2,3,4^ and are thus at risk for complications, including pneumonia, sepsis, barotrauma, and pulmonary edema,^5,6^ all of which can be associated with longer intensive care unit (ICU) and hospital stays, increased costs, and increased morbidity and mortality.^5,7^ To provide an objective measurement of ventilator-associated complications and to expand the previous subjective surveillance definitions of ventilator-associated pneumonia (VAP),^8^ in 2013 the Centers for Disease Control and Prevention (CDC) implemented a new surveillance definition for adults called ventilator-associated event (VAE), defined as sustained elevation in daily minimum positive end-expiratory pressure and/or fraction of inspired oxygen (Fio_2_) after a period of stability or improvement.^9^ In 2016, a multidisciplinary working group convened by the CDC, with representation from multiple societies, developed a modified definition of VAE for neonates and children termed pediatric VAE (PedVAE),^10^ defined as an increase in daily minimum Fio_2_ and/or mean arterial pressure (MAP) after a period of stability or improvement. This PedVAE definition was implemented by the CDC in January 2019 but does not include the tiered adult VAE definitions for infection-related ventilator-associated conditions and possible VAP.

Multicenter retrospective studies have shown that neonates and children with PedVAEs have longer lengths of ICU and hospital stays and increased mortality compared with those without PedVAE.^10,11^ Risk factors for PedVAEs include fluid overload in pediatric and cardiac ICUs, with 8-fold increased odds of PedVAEs for patients with the most fluid overload compared with those without any fluid overload.^12^ The same study found that weaning from sedation and ventilator support was associated with lower odds of PedVAEs in neonatal ICUs.^12^ However, little evidence exists on whether the reduction of these risk factors plays a role in prevention of PedVAEs.

The Children’s Hospitals’ Solutions for Patient Safety (SPS), a network of more than 140 hospitals in North America formally committed to improving patient safety in pediatrics, began tracking PedVAEs in most of its member hospitals in January 2017. A subset of those hospitals, called the Pioneer cohort, agreed to study 3 interventions (referred to as test factors) as possible future elements of a bundled intervention (ie, care bundle) to prevent PedVAEs. These test factors included performance of a multidisciplinary apparent cause analysis (ACA) for every PedVAE, daily discussion of extubation readiness, and daily discussion of fluid balance goals. In this quality improvement study, we aimed to assess whether adherence to 1 or more test factors was associated with a reduction in PedVAE rates. With this intervention, we aimed to decrease by 20% the rate of PedVAE per 1000 ventilator days among the Pioneer cohort by December 2020.

## Methods

The Cincinnati Children’s Hospital Medical Center Institutional Review Board deemed this quality improvement study exempt from review and waived the informed consent requirement because the data used were deidentified. We followed the Standards for Quality Improvement Reporting Excellence (SQUIRE) reporting guideline.^13^

### Study Design and Bundle Development

The study design was modeled after the recommended approach by the SPS network for Pioneer cohorts, described by Lyren and colleagues,^14^ for quality improvement bundle development: a leadership team selects interventions (test factors) to try and then guides the work during the Pioneer process. Participating hospitals then implement the test factors with a high degree of reliability. Subsequent analysis evaluates whether reliable implementation of that test factor is associated with the desired result.

In April 2017, a 2-day VAE strategy meeting of the SPS staff and PedVAE Study Group investigators was held to discuss PedVAE prevention, construct a key driver diagram (eFigure 1 in Supplement 1), define the test factors (including a measurement plan), and establish the timeline for test factor implementation and monitoring. Risk factors for PedVAEs were identified in a previous publication.^12^ Based on the previous study^12^ and the strategy meeting, the proposed quality improvement bundle consisted of 3 test factors: multidisciplinary ACA of every PedVAE, daily discussion of extubation readiness, and daily discussion of fluid balance goals (Box). Multidisciplinary ACA is a standard test factor in the Pioneer cohort work.^14^ The other 2 test factors were chosen based on findings from a 2017 study that risk factors for PedVAEs included positive fluid balance and sedation-weaning practices.^12^

Box. **Quality Improvement Bundle of 3 Test Factors and Definitions**

**Multidisciplinary ACA**

1. A multidisciplinary ACA event form completed for each event
2. ACA used to inform Pareto charts of institutional-specific causes of PedVAE to identify areas for improvement

**Daily Discussion of Extubation Readiness**

1. Discussion conducted on all individual patients weaning toward extubation
2. Discussion includes
3. Necessity for ETT
4. Target extubation time
5. Respiratory support plan
6. Pre-extubation sedation, analgesic, or restraints plan
7. Postextubation sedation or analgesic plan
8. Scheduled reevaluation time
9. Discussion attended by at least RN, RT, and MD or NP or PA
10. Discussion conducted once or twice daily

**Daily Discussion of Fluid Balance Goals**

1. Identification and discussion of patient-specific fluid balance goals for patients receiving invasive mechanical ventilation
2. Fluid balance goal documented at least daily
3. Discussion conducted once or twice daily

Abbreviations: ACA, apparent cause analysis; ETT, endotracheal tube; MD, doctor of medicine; NP, nurse practitioner; PA, physician assistant; PedVAE, pediatric ventilator-associated event; RN, registered nurse; and RT, respiratory therapist.

### Operational Definition and Participants

Previously, PedVAE was defined as a sustained worsening in oxygenation (ie, increase in daily minimum Fio_2_ of ≥0.25 or daily minimum MAP ≥4 mm Hg for at least 2 calendar days) after a baseline period of stability or improvement (ie, ≥2 calendar days of stable or decreasing daily minimum Fio_2_ or MAP).^10^ The study population included patients who were mechanically ventilated with an endotracheal tube or tracheostomy tube and cared for in a cardiac or neonatal ICU of an SPS network hospital.

Ninety-five hospitals in the SPS network across North America started recording PedVAE data in January 2017. Twenty-one hospitals, called the Pioneer cohort, volunteered to participate in a collaborative PedVAE improvement initiative. The remaining 74 hospitals that recorded PedVAE rates but did not perform further PedVAE improvement under SPS network guidance were deemed as the non-Pioneer cohort.

The ACA measurement plan was launched in January 2018. The ACA is a process in quality improvement that involves an investigation of a safety event. It is usually completed by local leaders in a discrete work setting and is less extensive than a root cause analysis.^15^ Many SPS network hospitals’ electronic health records (EHRs) were not yet equipped with the tools needed to detect PedVAEs and/or record ACA information at the time of the measurement plan launch. Standardized manual audits of patients who received ventilation were performed if electronic surveillance was not available. The test factors of daily discussion of extubation readiness and daily discussion of fluid balance goals were defined and distributed to the Pioneer cohort in July 2018. Standardized audits of nursing, respiratory therapy, and/or physician documentation in the EHR were conducted to evaluate compliance with test factors.

Training by the PedVAE Study Group leaders was performed with relevant teams during quarterly webinars and in-person SPS network–wide National Learning Sessions (NLSs). Webinars involved data presentations by individual hospitals, shared learnings between hospitals, and question-and-answer sessions with leaders and subject matter experts. The NLSs focused on the PedVAE definition in May 2018 and on implementation of the test factors in May 2019. Topics at the NLSs included education and discussion of the challenges surrounding test factor implementation. Hospitals in the Pioneer cohort were encouraged to start the test factor implementation when their teams had secured sufficient data analytic support and buy-in from their local champions (ie, physicians, nurses, and administration with authority to make practice changes at their respective hospitals).

### Data Submission

Each SPS network hospital was instructed to submit monthly outcome data for an 8-month baseline period (January 1 to August 31, 2017) and a 33-month implementation (postbaseline) period (September 1, 2017, to May 31, 2020). A separate study period (July 1, 2018, to May 31, 2020) was defined as the time after the test factors were distributed to the Pioneer cohort. Hospitals in both the Pioneer and non-Pioneer cohorts that submitted outcomes data for at least 80% of the baseline period and at least 80% of the postbaseline period were considered to be reliable and were included in the analyses. Thus, 9 Pioneer and 43 non-Pioneer hospitals were excluded from the analyses.

All hospitals in the Pioneer and non-Pioneer cohorts submitted monthly outcome data. The required data elements included the number of total PedVAEs for that month, the number of ventilator days, and the ICU (pediatric, cardiac, or neonatal) in which the PedVAE occurred.

Pioneer cohort hospitals submitted monthly data for each test factor. Compliance to a test factor was defined as following each test factor’s definition, as shown in the Box. The required data elements for the test factors included indicating whether each test factor was implemented that month and, if so, the total number of patients audited, the number of patients to which the test factor was not applicable, the number of patients for whom the test factor was used correctly, and the number of patients for whom the test factor was used incorrectly. Hospitals were asked to complete 20 audits per month for the daily discussion of extubation readiness test factor and 30 audits per month for the daily discussion of fluid balance goals test factor.

Pioneer cohort hospitals were asked to implement all test factors in all neonatal, cardiac, and pediatric ICUs. These hospitals used a variety of implementation methods, including high-reliability strategies that ranged from education to real-time weekly or monthly audits of compliance with daily discussion of extubation readiness and/or fluid balance goals during rounds or in clinical documentation. For the ACA data, a standardized template (eAppendix in Supplement 1) created by the SPS network was adapted as needed by participating hospitals. The ACA information was collected on paper or electronically via REDCap (Vanderbilt University) based on institutional preference.

### Statistical Analysis

As recommended by the SPS network^14^ for the Pioneer cohort quality improvement approach, Shewhart control U-charts were used to explore patterns in the monthly PedVAE outcomes over the baseline and postbaseline periods. Improvement was defined as a series of 8 or more data points on the same desired side of the centerline and that result in a centerline shift, which is a standard rule for special cause determination on Shewhart control charts.^16^ The centerline was determined by calculating the mean PedVAE rate during the baseline period, providing an 8-month baseline, which has been used in a previous SPS network study.^17^ The 2 test factors selected a priori for analysis were daily discussion of extubation readiness and daily discussion of fluid balance goals. Each test factor was analyzed independently at the network level to understand the degree to which reliable implementation of each factor changed the overall results. To aid in this analysis, hospital data were placed in performance groups similar to those described by Lyren et al.^14^ The 3 performance groups were (1) measuring the process for 50% or more of the study period and performing with 80% or greater reliability, (2) measuring the process for 50% or more of the study period but performing with less than 80% reliability, and (3) not measuring the process or reporting less than 50% of the study period.

The mean PedVAE rates during the baseline period (January to August 2017) and after test factors were distributed to the Pioneer cohort (July 2018 to May 2020) were ascertained. Control charts were used to study the association between the PedVAE outcome rates and reliability to a test factor for each hospital. Unpaired, 2-tailed *t* tests were used to compare PedVAE rates before and after centerline shifts. Two-sided *P* < .05 was considered significant.

In post hoc analyses, we constructed Shewhart control U-charts to plot the PedVAE rates by reliability in reporting ACA data. Hospitals were deemed as reliable reporters if they submitted at least 80% of their PedVAE data from 2017 to 2020 and had greater than 80% reliability for at least 2 of these 4 years. Hospitals were deemed as nonreliable reporters if they submitted less than 80% of their PedVAE data in the same timeframe or had less than 80% reliability for at least 2 of the 4 years. Therefore, the nomenclature of the 3 reliability groups for the other 2 test factors were not used for the ACA data. Analyses were performed between September 2021 and April 2023, using SAS Enterprise Guide 8.1 (SAS Institute Inc), and Microsoft Excel for Microsoft 365 MSO (Microsoft Corp).

## Results

Twelve of the 21 hospitals (57%) in the Pioneer cohort were considered to be reliable reporters of PedVAE outcome data and continued submitting data every month through May 2020 (eFigure 2 in Supplement 1), encompassing a total of 405 851 ventilator days. The other 9 Pioneer cohort hospitals were excluded from the analyses. For the 12 Pioneer cohort hospitals included in the analyses, the total number of ventilator days in each hospital ranged from 5525 to 119 588, with a median (IQR) of 25 927 (17 371-37 243) days per hospital. Among the 74 hospitals in the non-Pioneer cohort, 33 (45%) were considered to be reliable reporters and contributed 735 691 ventilator days. In the non-Pioneer cohort, ventilator days per hospital ranged from 1990 to 84 897 days, with a median (IQR) of 15 832 (9258-27 484) days. All 12 Pioneer cohort hospitals that were reliable reporters of PedVAE rate data also submitted data on daily discussion of extubation readiness or daily discussion of fluid balance goals. The median (IQR) number of audits per month per hospital was 26 (11-69) for daily discussion of extubation readiness and 32 (30-51) for daily discussion of fluid balance goals. Eleven of these 12 hospitals (92%) submitted ACA data.

### Aggregate PedVAE Rates

**Figure 1.**
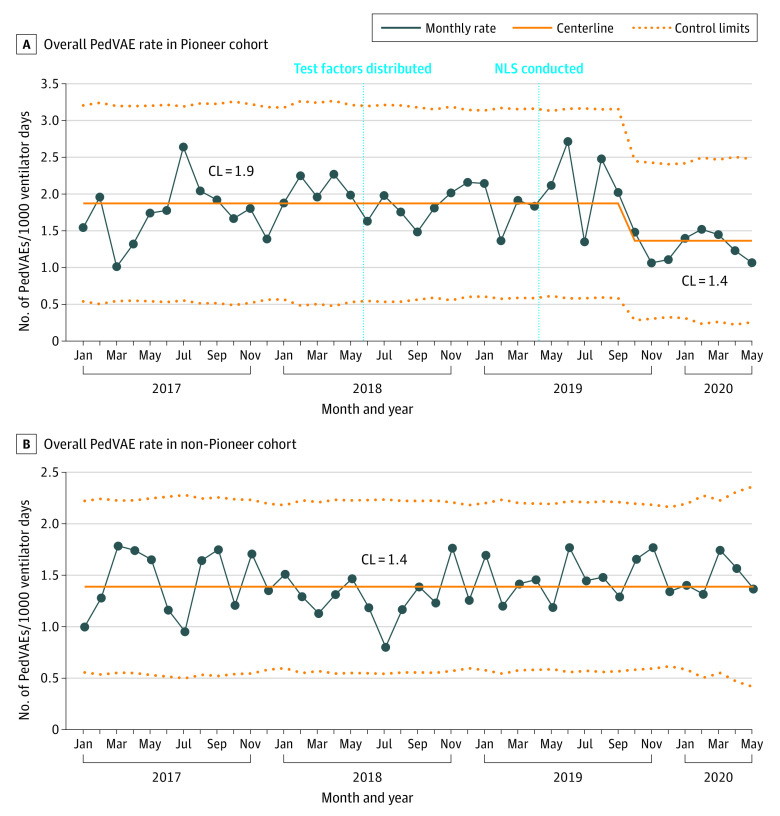
Shewhart Control U-Charts of the Overall Pediatric Ventilator-Associated Event (PedVAE) Rates Among Study Cohorts A, The Pioneer cohort comprised 12 hospitals that received dedicated training and resources to reduce the incidence of PedVAE. B, The non-Pioneer cohort comprised 33 hospitals that did not receive dedicated training and resources. CL indicates centerline; NLS, National Learning Session.

The Shewhart control U-chart for the 12 reliable reporters in the Pioneer cohort is shown in Figure 1A, and it demonstrates a centerline shift. This shift occurred in September 2019, 5 months after the NLSs in May 2019, when focused education on the extubation readiness and fluid management test factors was provided. The aggregate PedVAE rate was 1.9 events per 1000 ventilator days before the centerline shift beginning in September 2019 and was 1.4 events per 1000 ventilator days after September 2019, amounting to an absolute rate difference of −0.6 (95% CI, −0.5 to −0.7; *P* < .001). For comparison, the control U-chart for 33 reliable reporters in the non-Pioneer cohort is shown in Figure 1B, and it does not show special cause variation. In the Pioneer cohort hospitals, 30-day mortality after PedVAEs ranged from 18% to 32% by ICU type, with the rate being lowest in the pediatric ICU (60% [71 of 118]) and highest in the neonatal ICU (72% [146 of 203]) (eTable in Supplement 1). Overall, there was a 26% reduction in PedVAEs.

### Test Factor Analyses

**Figure 2.**
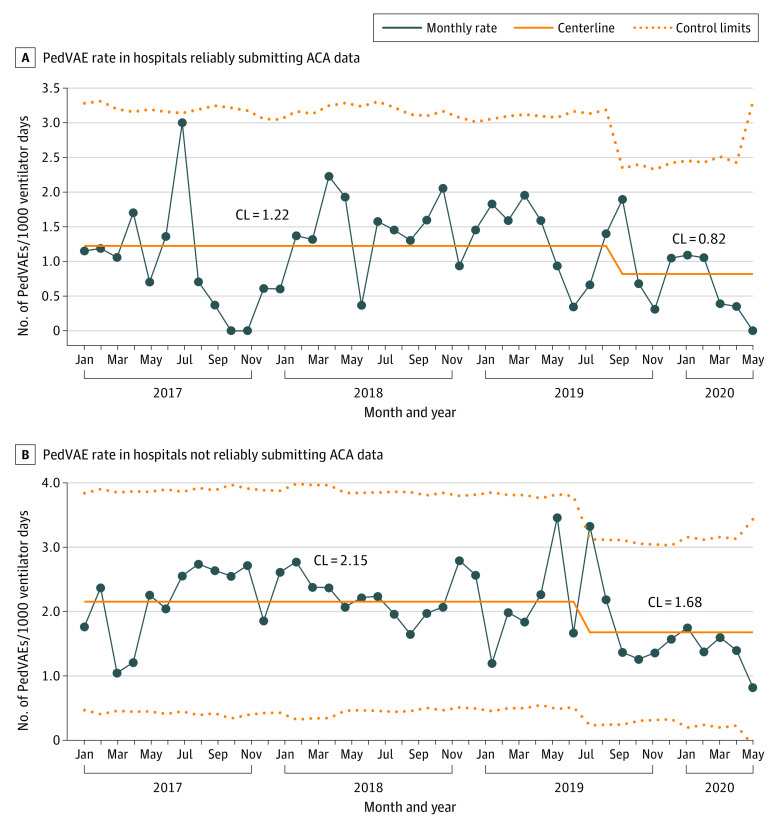
Shewhart Control U-Charts of Pioneer Cohort Hospitals Grouped by Reliability With Submitting Apparent Cause Analysis (ACA) Data A, Four hospitals reliably reported ACA data by submitting at least 80% of pediatric ventilator-associated events (PedVAEs) per calendar year and PedVAE outcome data. B, Seven hospitals did not reliably report ACA data by submitting less than 80% of PedVAEs per calendar year and PedVAE outcome data. CL indicates centerline.

Four of 12 Pioneer cohort hospitals (33%) were considered to be reliable reporters of ACA data. Shewhart control U-charts for both the reliable and nonreliable reporters of ACA data demonstrated special cause variation beginning in the latter half of 2019 (Figure 2A and B). The centerline for the reliable reporters decreased from 1.22 to 0.82 events per 1000 ventilator days (33% decrease) in October 2019, and the centerline for the nonreliable reporters decreased from 2.15 to 1.68 events per 1000 ventilator days (23% decrease) in September 2019. The ACA-derived causes of PedVAE had commonalities among the 3 ICU types, with infection being the most common in the pediatric ICU (33%) and neonatal ICU (35%) and change in physiology being more common than infection in the cardiac ICU (40% vs 24%) (eFigure 4 in Supplement 1). The infection category included tracheitis, nonpulmonary sepsis, VAP, and hospital-onset respiratory viral infections.

**Figure 3.**
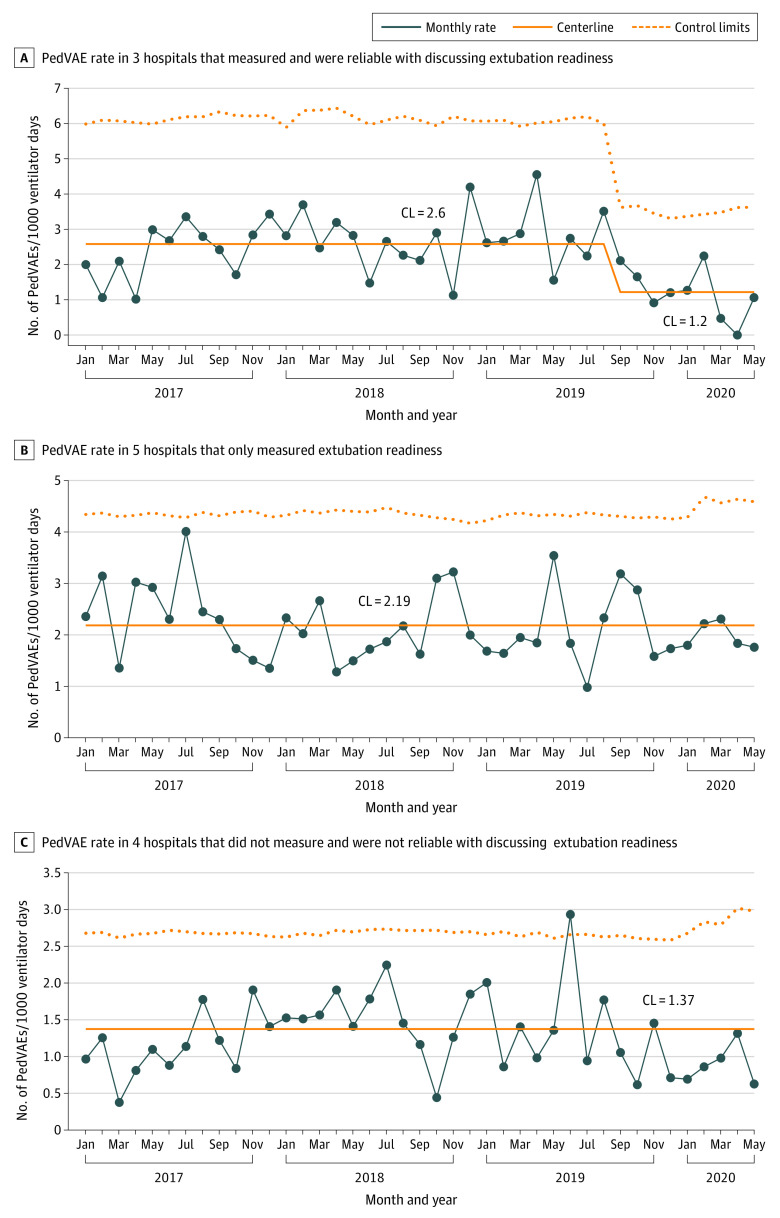
Shewhart Control U-Charts of Pioneer Cohort Hospitals Grouped by Reliability With Discussing Extubation Readiness Factor CL indicates centerline; PedVAE, pediatric ventilator-associated event.

Figure 3 shows the Shewhart control U-charts for each performance group of hospitals for the extubation readiness test factor. A centerline shift was found in the 3 hospitals that were considered to be measuring and reliable for the test factor, from 2.6 events per 1000 ventilator days to 1.2 events per 1000 ventilator days, amounting to a 54% decrease (absolute rate difference, −1.4; 95% CI, −1.0 to −1.7; *P* < .001).

Only 1 hospital was deemed as measuring and reliable for discussing the fluid balance goals test factor, and it showed evidence of special cause variation, with the PedVAE rate decreasing from 1.6 events per 1000 ventilator days to 0.6 events per 1000 ventilator days in September 2019 (absolute rate difference, −1.0; 95% CI, −0.1 to −1.9; *P* = .03) (eFigure 3A in Supplement 1). There was no evidence of special cause variation in the aggregate PedVAE rate of the 2 hospitals that were considered to be measuring only but not reliable, but there was such evidence in 9 hospitals deemed as not measuring, with the PedVAE rate decreasing from 2.0 events per 1000 ventilator days to 1.5 events per 1000 ventilator days in September 2019 (absolute rate difference, −0.5; 95% CI, −0.4 to −0.7; *P* < .001) (eFigure 3B and C in Supplement 1).

## Discussion

In this study, we presented the results from a large multicenter quality improvement initiative in the SPS network to decrease PedVAE rates by implementing test factors that were deemed promising by previous investigations and expert opinion. The 3 test factors were multidisciplinary ACAs, daily discussion of extubation readiness, and daily discussion of fluid balance goals. Hospitals in the Pioneer cohort that received training and education about PedVAE had a significant decrease in PedVAE rate, whereas the non-Pioneer cohort hospitals that did not receive the same interventions showed no change in PedVAE rate. The lower PedVAE rate in the Pioneer cohort did meet the PedVAE Study Group’s SMART (Specific, Measurable, Achievable, Relevant, and Time Bound) aim of decreasing the PedVAE rate by 20% by December 2020, as defined in the key driver diagram. On analysis of reliability in adhering to test factors, 3 hospitals that were most reliable to discuss the extubation readiness test factor demonstrated a decrease in aggregate PedVAE rate, coinciding with the decrease in PedVAE rate in the overall Pioneer cohort. In Pioneer cohort hospitals, PedVAE rates decreased regardless of hospitals’ reliability in submitting ACA data. The baseline PedVAE rate of the Pioneer cohort was higher than that of the non-Pioneer cohort and declined to the same baseline rate of the non-Pioneer cohort. Hospital characteristics that may explain this difference were not collected, and this difference may be a product of selection bias.

Data were limited regarding reliability with the fluid balance goal test factor, so its implication was unclear. Considering the 18% to 32% 30-day mortality in patients with PedVAEs, the 26% reduction in PedVAEs in the Pioneer cohort hospitals could be translated to a substantial reduction in mortality in critically ill patients who received mechanical ventilation and to widespread adoption of this proposed PedVAE prevention bundle.

The Institute for Healthcare Improvement has endorsed bundled care as an important tool in achieving optimal patient safety.^18^ Meta-analyses have reported reduced mortality associated with implementation of ventilator bundles in adult ICUs.^19^ Studies similar to the present study but conducted in single centers have found that reductions in pediatric VAP were associated with adherence to standardized bundles of quality improvement interventions, such as brushing teeth and oropharyngeal suctioning.^20,21,22,23^ Daily assessment of extubation readiness is recommended in the Society for Healthcare Epidemiology of America guidelines as an essential practice to reduce the incidence of VAP and VAE in adults and children, both within and outside the neonatal period.^24^ These guidelines specifically include conducting spontaneous breathing trials in patients without contraindications, minimizing sedation, taking steps to reduce reintubation risk, and using caffeine for apnea of prematurity within 72 hours of birth.^24^ Additionally, the test factors used in this study did not include traditional VAP bundle elements, which have been suggested in recent single-center studies to be associated with decreased PedVAE rates and infection-associated PedVAE.^25,26^ However, PedVAE and pediatric VAP are distinct entities.^27^ Patients with more severe illness are included in the PedVAE definition compared with those identified by the prior VAP definitions. The relative contributions of infection vs fluid overload in deterioration on the ventilator are complex; thus, the most important prevention elements likely also vary by cause of PedVAE.^28,29^

Given that performing ACAs could help clinicians identify potential causes or risk factors of PedVAEs, reporting and discussing these ACAs could lead to greater attention to the event and its prevention. Increased reporting in root cause analyses and ACAs have been associated with improved patient outcomes.^30,31,32^ In the present study, however, both reliable and nonreliable reporters of ACA data showed a centerline shift in PedVAE rates. This shift may be associated with the PedVAE education that was provided to both the reliable and nonreliable reporters of ACA data.

Results of this study showed a long delay between the requested initiation of the intervention and the centerline shift in aggregate PedVAE rate among the 12 Pioneer cohort hospitals. Specifically, the test factors were started in July 2018, and a shift in PedVAE rate among the 12 Pioneer cohort hospitals did not occur until September 2019. This finding was likely associated with hospitals having varying start times for their full implementation of the test factors, often many months afterward. We hypothesized that the timing of the centerline shift in 2019 was associated with the NLS in May 2019, highlighting the importance and implications of the SPS network’s all teach, all learn approach for quality improvement during these learning sessions.

### Limitations

This study has limitations. As in any quality improvement initiative with before-and-after comparisons, unmeasured variables might confound the results. Analysis of data by unit was not possible due to lack of unit-specific data during the baseline period. Few hospitals were reliable with the fluid balance goal test factor, likely because this was a new process in these hospitals. In contrast, many hospitals already received training and education on the extubation readiness test factor from their previous participation in the unplanned extubation prevention initiative of the SPS network.^17^ Additionally, the accuracy of the submitted PedVAE rates data was not verified. Selection bias was potentially introduced into the analysis when filtering the analysis to include the hospitals that were most reliable. Due to the nature of the study design, it was impossible to definitively identify a causal association between test factors and decrease in PedVAE rates.

## Conclusions

Implementation of a quality improvement intervention in multiple hospitals across North America was followed by a 26% decrease in PedVAE rate among hospitals that received training and education on PedVAE prevention. Hospitals that reliably discussed extubation readiness had a significant decrease in PedVAE rate compared with hospitals that did not reliably implement this test factor. Moving forward, we recommend that ICU teams seeking to reduce their burden of PedVAEs incorporate daily discussion of extubation readiness during morning rounds.

## Notes

Supplement 1. **eTable.** ACA-Reported Mortality Within 30 Days of PedVAE by ICU Type **eFigure 1.** Key Driver Diagram (KDD) Displaying the SMART Aim, Key Drivers, and Factors in the Intervention **eFigure 2.** Flowchart Demonstrating Process of Identifying Hospitals To Be Included in Final Analysis **eFigure 3.** Shewhart Control U-Charts of Hospitals Grouped by Reliability to the Fluid Balance Goal Test Factor **eFigure 4.** Suspected Causes of PedVAE by ICU Type Based on ACA Reports **eAppendix.** ACA Survey Template

Supplement 2.
Nonauthor Collaborators

Supplement 3.
Data Sharing Statement

## References

[1] Wunsch H, Linde-Zwirble WT, Angus DC, Hartman ME, Milbrandt EB, Kahn JM. The epidemiology of mechanical ventilation use in the United States. Crit Care Med. 2010;38(10):1947-1953. doi:10.1097/CCM.0b013e3181ef4460 20639743

[2] Principi T, Fraser DD, Morrison GC, . Complications of mechanical ventilation in the pediatric population. Pediatr Pulmonol. 2011;46(5):452-457. doi:10.1002/ppul.21389 21194139

[3] Rubinson L, Vaughn F, Nelson S, . Mechanical ventilators in US acute care hospitals. Disaster Med Public Health Prep. 2010;4(3):199-206. doi:10.1001/dmp.2010.18 21149215

[4] Wunsch H, Wagner J, Herlim M, Chong DH, Kramer AA, Halpern SD. ICU occupancy and mechanical ventilator use in the United States. Crit Care Med. 2013;41(12):2712-2719. doi:10.1097/CCM.0b013e318298a139 23963122 PMC3840149

[5] Kahn JM, Goss CH, Heagerty PJ, Kramer AA, O’Brien CR, Rubenfeld GD. Hospital volume and the outcomes of mechanical ventilation. N Engl J Med. 2006;355(1):41-50. doi:10.1056/NEJMsa053993 16822995

[6] Rubenfeld GD, Caldwell E, Peabody E, . Incidence and outcomes of acute lung injury. N Engl J Med. 2005;353(16):1685-1693. doi:10.1056/NEJMoa050333 16236739

[7] Behrendt CE. Acute respiratory failure in the United States: incidence and 31-day survival. Chest. 2000;118(4):1100-1105. doi:10.1378/chest.118.4.1100 11035684

[8] Centers for Disease Control and Prevention. Pneumonia (ventilator-associated [VAP] and non–ventilator-associated pneumonia [PNEU]) event. 2021. Accessed November 2022. https://www.cdc.gov/nhsn/pdfs/pscmanual/6pscvapcurrent.pdf

[9] Klompas M. Complications of mechanical ventilation—the CDC’s new surveillance paradigm. N Engl J Med. 2013;368(16):1472-1475. doi:10.1056/NEJMp1300633 23594002

[10] Cocoros NM, Kleinman K, Priebe GP, ; Pediatric Ventilator-Associated Conditions Study Team. Ventilator-associated events in neonates and children—a new paradigm. Crit Care Med. 2016;44(1):14-22. doi:10.1097/CCM.0000000000001372 26524075 PMC10884951

[11] Cocoros NM, Priebe GP, Logan LK, . A pediatric approach to ventilator-associated events surveillance. Infect Control Hosp Epidemiol. 2017;38(3):327-333. doi:10.1017/ice.2016.277 27917737 PMC13262793

[12] Cocoros NM, Priebe G, Gray JE, . Factors associated with pediatric ventilator-associated conditions in six U.S. hospitals: a nested case-control study. Pediatr Crit Care Med. 2017;18(11):e536-e545. doi:10.1097/PCC.0000000000001328 28914722 PMC13262792

[13] Ogrinc G, Davies L, Goodman D, Batalden P, Davidoff F, Stevens D. SQUIRE 2.0 (Standards for QUality Improvement Reporting Excellence): revised publication guidelines from a detailed consensus process. BMJ Qual Saf. 2016;25(12):986-992. doi:10.1136/bmjqs-2015-004411 26369893 PMC5256233

[14] Lyren A, Dawson A, Purcell D, Hoffman JM, Provost L. Developing evidence for new patient safety bundles through multihospital collaboration. J Patient Saf. 2021;17(8):e1576-e1584. doi:10.1097/PTS.0000000000000564 30720545

[15] Crandall KM, Sten MB, Almuhanna A, Fahey L, Shah RK. Improving apparent cause analysis reliability: a quality improvement initiative. Pediatr Qual Saf. 2017;2(3):e025. doi:10.1097/pq9.0000000000000025 30229162 PMC6132456

[16] Montgomery DC. Introduction to Statistical Quality Control. John Wiley & Sons; 2007.

[17] Klugman D, Melton K, Maynord PON, . Assessment of an unplanned extubation bundle to reduce unplanned extubations in critically Ill neonates, infants, and children. JAMA Pediatr. 2020;174(6):e200268. doi:10.1001/jamapediatrics.2020.0268 32282029 PMC7154960

[18] Resar RGF, Haraden C, Nolan TW. Using Care Bundles to Improve Health Care Quality. Institute for Healthcare Improvement; 2012.

[19] Pileggi C, Mascaro V, Bianco A, Nobile CGA, Pavia M. Ventilator bundle and its effects on mortality among ICU patients: a meta-analysis. Crit Care Med. 2018;46(7):1167-1174. doi:10.1097/CCM.0000000000003136 29629985

[20] Hutchins K, Karras G, Erwin J, Sullivan KL. Ventilator-associated pneumonia and oral care: a successful quality improvement project. Am J Infect Control. 2009;37(7):590-597. doi:10.1016/j.ajic.2008.12.007 19716460

[21] Kelleghan SI, Salemi C, Padilla S, . An effective continuous quality improvement approach to the prevention of ventilator-associated pneumonia. Am J Infect Control. 1993;21(6):322-330. doi:10.1016/0196-6553(93)90390-P 8122805

[22] Dubose J, Teixeira PGR, Inaba K, . Measurable outcomes of quality improvement using a daily quality rounds checklist: one-year analysis in a trauma intensive care unit with sustained ventilator-associated pneumonia reduction. J Trauma. 2010;69(4):855-860. doi:10.1097/TA.0b013e3181c4526f 20032792

[23] de Neef M, Bakker L, Dijkstra S, Raymakers-Janssen P, Vileito A, Ista E. Effectiveness of a ventilator care bundle to prevent ventilator-associated pneumonia at the PICU: a systematic review and meta-analysis. Pediatr Crit Care Med. 2019;20(5):474-480. doi:10.1097/PCC.0000000000001862 31058785

[24] Klompas M, Branson R, Cawcutt K, . Strategies to prevent ventilator-associated pneumonia, ventilator-associated events, and nonventilator hospital-acquired pneumonia in acute-care hospitals: 2022 update. Infect Control Hosp Epidemiol. 2022;43(6):687-713. doi:10.1017/ice.2022.88 35589091 PMC10903147

[25] Wu ALR, Bullock KJ, Ormsby J, . Kamishibai card rounding to prevent infection-related pediatric ventilator-associated events. Paper presented at: 2022 Critical Care Congress, Society of Critical Care Medicine; February 2022; San Juan, Puerto Rico.

[26] Wu ALR, Bullock KJ, Ormsby J, Yuen JC, Sandora TJ, Priebe GP. Using Kamishibai cards to prevent pediatric ventilator associated events: a single center study. Paper presented at: 2023 Critical Care Congress, Society of Critical Care Medicine; January 2023; San Francisco, California.

[27] Ziegler KM, Haywood JD, Sontag MK, Mourani PM. Application of the new Centers for Disease Control and Prevention surveillance criteria for ventilator-associated events to a cohort of PICU patients identifies different patients compared with the previous definition and physician diagnosis. Crit Care Med. 2019;47(7):e547-e554. doi:10.1097/CCM.0000000000003766 30985451 PMC7089756

[28] Papakyritsi D, Iosifidis E, Kalamitsou S, . Epidemiology and outcomes of ventilator-associated events in critically ill children: evaluation of three different definitions. Infect Control Hosp Epidemiol. 2023;44(2):216-221. doi:10.1017/ice.2022.97 35506391

[29] Sick-Samuels AC, Priebe GP. Optimizing surveillance for pediatric ventilator-associated events—but are they preventable? Infect Control Hosp Epidemiol. 2023;44(2):175-177. doi:10.1017/ice.2022.121 35611848 PMC9691785

[30] Shah F, Falconer EA, Cimiotti JP. Does root cause analysis improve patient safety? a systematic review at the Department of Veterans Affairs. Qual Manag Health Care. 2022;31(4):231-241. doi:10.1097/QMH.0000000000000344 35170581

[31] Kandil SB, Emerson BL, Hooper M, . Reducing unplanned extubations across a children’s hospital using quality improvement methods. Pediatr Qual Saf. 2018;3(6):e114. doi:10.1097/pq9.0000000000000114 31334446 PMC6581473

[32] Kaplan S, Maydick-Youngberg D, Liao J, Francis K. Journey to zero harm. Nurs Manage. 2019;50(11):52-54. doi:10.1097/01.NUMA.0000602812.42313.f2 31688548

