# Supplementary material for: Pediatric Ventilator-Associated Events Before and After a Multicenter Quality Improvement Initiative
Source: JAMA Netw Open. 2023 Dec 7;6(12):e2346545. doi: 10.1001/jamanetworkopen.2023.46545 (PMC10704274; doi:10.1001/jamanetworkopen.2023.46545)
Supplement: Supplement 1. — eTable. ACA-Reported Mortality Within 30 Days of PedVAE by ICU Type eFigure 1. Key Driver Diagram (KDD) Displaying the SMART Aim, Key Drivers, and Factors in the Intervention eFigure 2. Flowchart Demonstrating Process of Identifying Hospitals To Be Included in Final Analysis eFigure 3. Shewhart Control U-Charts of Hospitals Grouped by Reliability to the Fluid Balance Goal Test Factor eFigure 4. Suspected Causes of PedVAE by ICU Type Based on ACA Reports eAppendix. ACA Survey Template [file jamanetwopen-e2346545-s001.pdf]

Supplemental Online Content

Wu AG, Madhavan G, Deakins K, et al; Solutions for Patient Safety PedVAE Study Group. Pediatric ventilator-associated events before and after a multicenter quality improvement initiative. *JAMA Netw Open*. 2023;6(12):e2346545. doi:10.1001/jamanetworkopen.2023.46545

eTable. ACA-Reported Mortality Within 30 Days of PedVAE by ICU Type

eFigure 1. Key Driver Diagram (KDD) Displaying the SMART Aim, Key Drivers, and Factors in the Intervention

eFigure 2. Flowchart Demonstrating Process of Identifying Hospitals To Be Included in Final Analysis

eFigure 3. Shewhart Control U-Charts of Hospitals Grouped by Reliability to the Fluid Balance Goal Test Factor

eFigure 4. Suspected Causes of PedVAE by ICU Type Based on ACA Reports

eAppendix. ACA Survey Template

This supplemental material has been provided by the authors to give readers additional information about their work.

© 2023 Wu AG et al. *JAMA Network Open*

eTable. ACA-reported mortality within 30 days of PedVAE by ICU type.

	PICU	CICU	NICU
Died	31% (36/118)	23% (18/77)	17% (34/203)
Survived 30 days or to hospital discharge/transfer	60% (71/118)	64% (49/77)	72% (146/203)
Unable to obtain data	9% (11/118)	13% (10/77)	11% (23/203)

PICU = pediatric intensive care unit. CICU = cardiac intensive care unit. NICU = neonatal intensive care unit

© 2023 Wu AG et al. *JAMA Network Open*

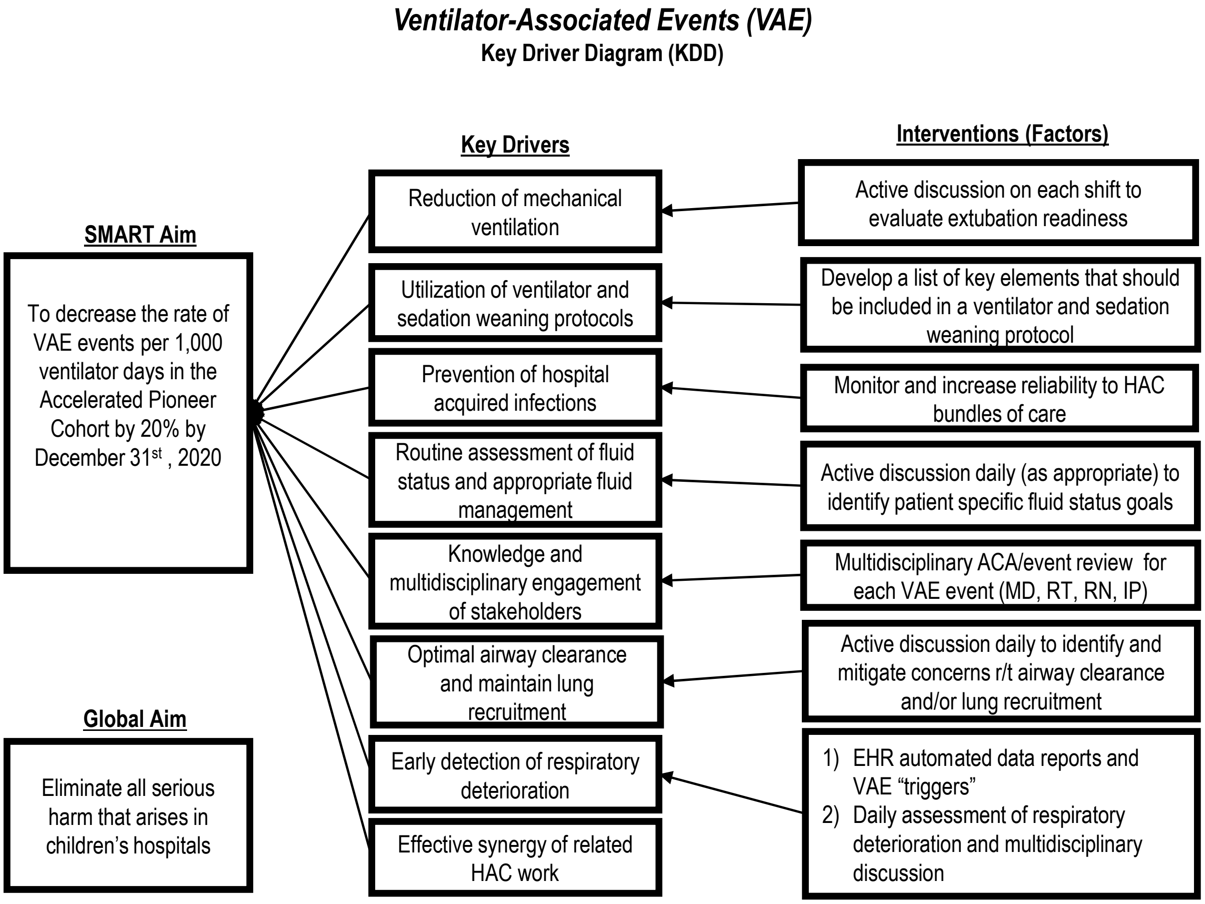

eFigure 1. Key Driver Diagram (KDD) displaying the SMART aim, key drivers, and factors in the intervention.

The KDD was developed during a 2-day meeting PedVAE Strategy Meeting hosted by SPS in April 2017 and attended by 15 participants including SPS clinical leaders (A. Lyren, M Coffey), SPS quality manager (L. Mustin), an SPS data analyst (G. Madhavan), two SPS project coordinators, a CDC representative, a SHEA representative, 6 pediatric intensivists (from NICU, CICU, and PICU), two infectious disease physicians, two ICU nurse specialists (from NICU and PICU), and respiratory therapists. Four of the participants were from 3 of the Pioneer Cohort hospitals.

Abbreviations: HAC = hospital acquired condition. ACA = apparent cause analysis. PedVAE = pediatric ventilator associated event

© 2023 Wu AG et al. *JAMA Network Open*

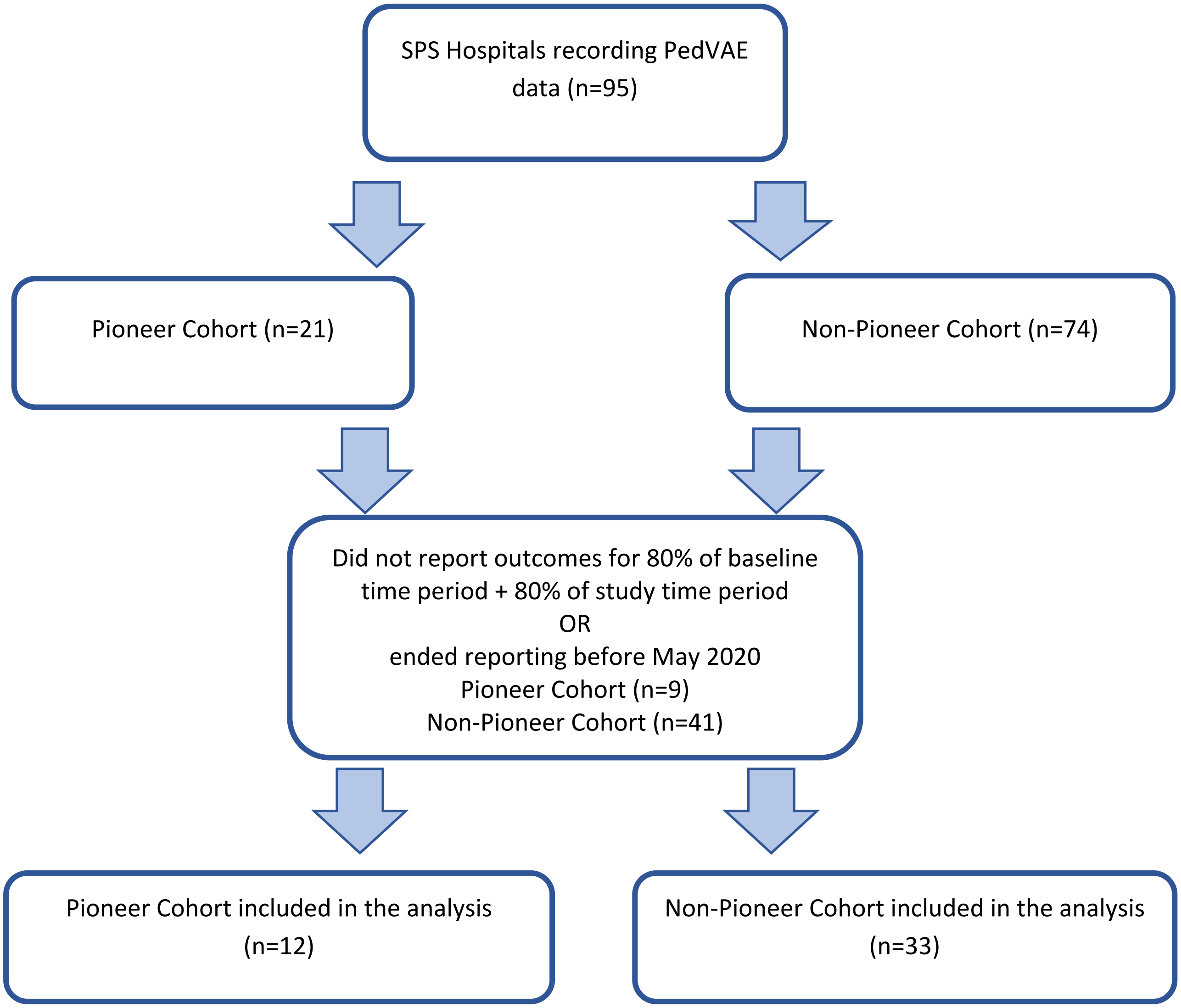

eFigure 2. Flowchart demonstrating process of identifying hospitals to be included in final analysis.

SPS = Solutions for Patient Safety. PedVAE = Pediatric ventilator-associated events.

© 2023 Wu AG et al. *JAMA Network Open*

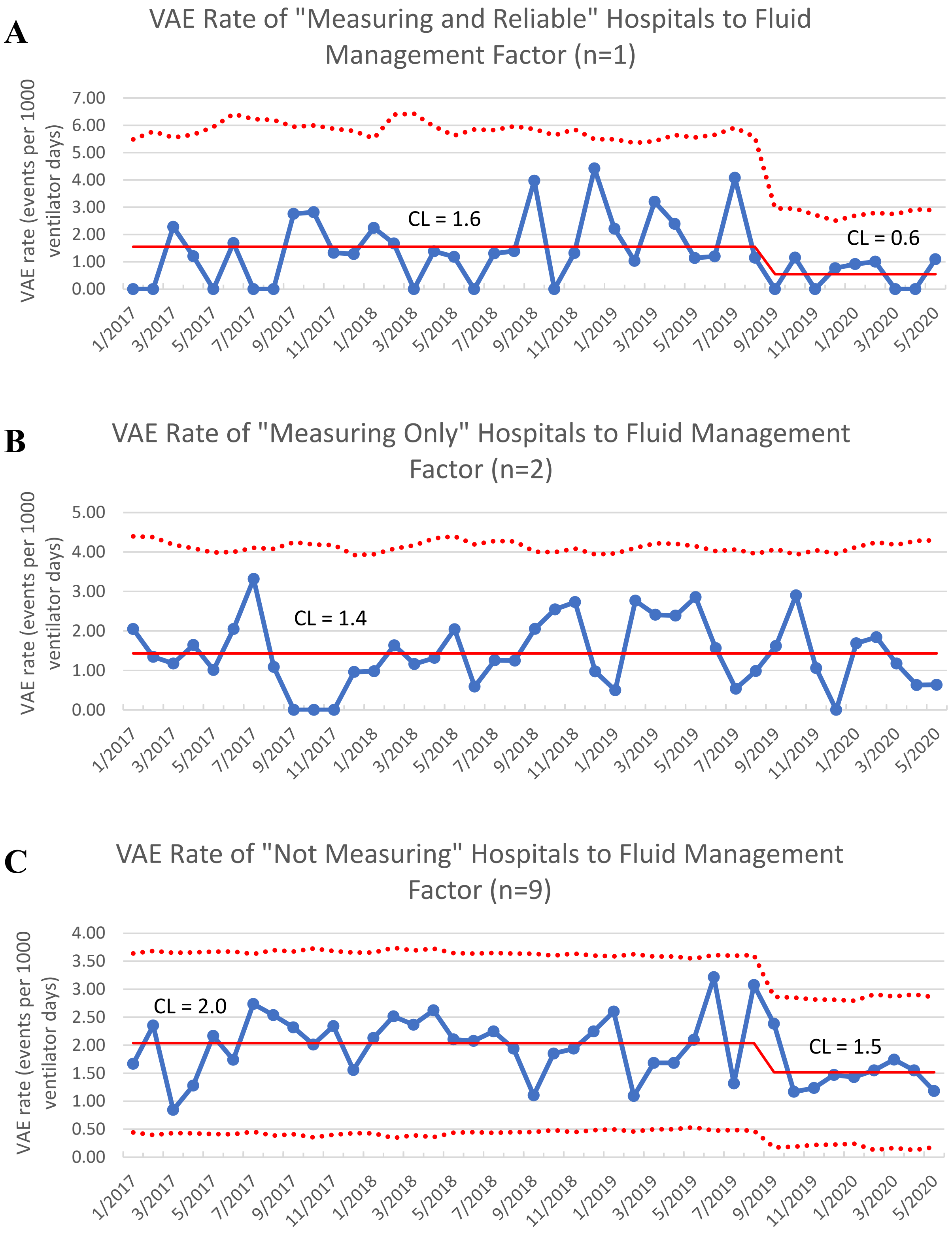

© 2023 Wu AG et al. *JAMA Network Open*

eFigure 3. Shewhart control U-charts of hospitals grouped by reliability to the fluid balance goal test factor.

A. Shewhart control U-chart for one hospital that was “measuring and reliable” for the fluid balance goal test factor. B. Shewhart control U-chart for hospitals who were “measuring only” for the fluid balance goal test factor. C. Shewhart control U-chart for hospitals who were “not measuring” for the fluid balance goal test factor. PedVAE = pediatric ventilator associated event. CL = centerline

© 2023 Wu AG et al. *JAMA Network Open*

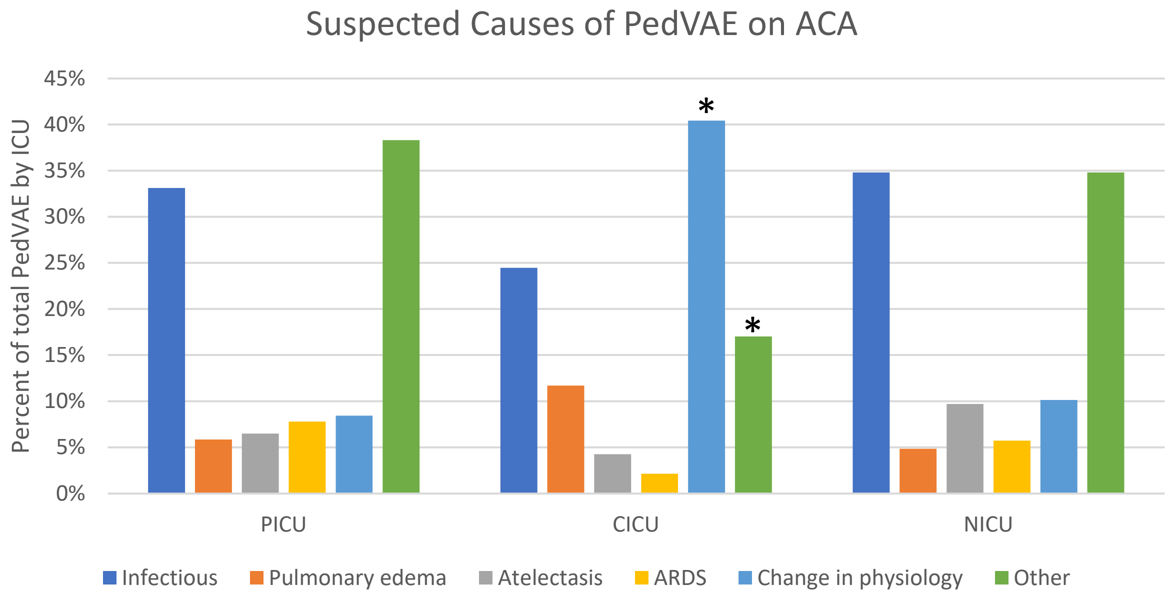

eFigure 4. Suspected causes of PedVAE by ICU type based on ACA reports.

Data represent ACA reports performed by 11 of the 12 Pioneer Cohort hospitals. Total ACA reports among PICU was 118; among CICU was 77; and among NICU was 203. Total percentages are more than 100% since more than one cause could be checked per report. The category "Other" provided a free text field, which included pulmonary hemorrhage, pulmonary hypertension, tracheobronchomalacia, exacerbation of chronic lung disease/bronchopulmonary dysplasia, fluid overload, cardiac surgery, closure/opening of chest or abdomen, congenital diaphragmatic hernia, and many other patient-specific possible etiologies. *p<0.001 by chi-squared tests in comparison to NICU and PICU. ACA = apparent cause analysis. PedVAE = pediatric ventilator associated event. PICU = pediatric intensive care unit. CICU = cardiac intensive care unit. NICU = neonatal intensive care unit. ARDS = acute respiratory distress syndrome.

© 2023 Wu AG et al. *JAMA Network Open*

eAppendix. ACA survey template

PedVAE ACA Data Collection Tool - Key Elements

At the time of VAE, did the patient have	◯ ETT<br>◯ Trach
Did the patient have a mortality within 30 days of the VAE criteria?	◯ Yes<br>◯ No (or hospital discharge or transfer prior to 30days)<br>◯ Unable to obtain the information
Which VAE Criteria were met?	◯ Increase in daily minimum* FiO2 of >= 0.25 (25 points) over the daily minimum FiO2 in the baseline period, sustained for >= 2 calendar days.<br>◯ Increase in daily minimum* MAP values of >= 4 cmH2O over the daily minimum MAP in the baseline period, sustained for >= 2 calendar days<br>◯ Both MAP and FiO2 criteria were met

Suspected Etiology/Contributing Factors

Suspected etiology of the VAE event (ex. Pneumonia, pulmonary edema, atelectasis, ARDS, etc.):	☐ Infectious (eg:Pneumonia, Viral infection, Sepsis etc.)<br>☐ Pulmonary edema<br>☐ Atelectasis<br>☐ ARDS<br>☐ Change in physiology (including cardiac surgery, closure/open of chest or abdomen, congenital diaphragmatic hernia repair)<br>☐ Other: Please describe
Please describe OTHER for "Suspected etiology"	__________________________________

© 2023 Wu AG et al. *JAMA Network Open*

Risk factors/Co-morbidity Diagnosis (factors that you/your team believes contributed to the risk of the VAE event):	________________________________

**Leading up to the event (Evaluate the 2 calendar days before VAE event)**	
Overall phase of patient's illness/care:	◯ Acute - overall care is escalating or static<br>◯ Weaning - patient is improving overall and care is de-escalating<br>◯ Chronic - long term/chronic patient where overall care is static
Was the patient receiving a neuromuscular blockade?	◯ Yes<br>◯ No
Was the patient fluid overloaded? (Pick 1)<br>We acknowledge this is subjective however, if your hospital decides to be more specific, you could calculate admission weight against weight on 2 calendar days before VAE event:<br>< 10% = Mild<br>10-20% = Moderate<br>> 20% = Severe	◯ Yes - Mild<br>◯ Yes - Moderate<br>◯ Yes - Severe<br>◯ No

**Since we are more interested on weight on day 0 and not weight on day -2 during the period of stability**<br><br>**We acknowledge this is subjective, SPS recommends you could calculate % fluid overload in one of two ways:**<br>**1. Weight-based (preferred, if weights available):**<br>**{[weight (kg) on day of VAE (day 0) minus weight (kg) on day -2]/[weight (kg) on day -2]}x100%**<br>**2. Fluid balance-based**<br>**{[cumulative fluid balance on day -1 and day -2 (liters)]/[weight (kg) on day -2, or most recent weight before day -2, or admission weight if that is the only weight available]}x100%**	
%Fluid overload	◯ Very Mild (< 5%)<br>◯ Mild (5-9%)<br>◯ Moderate (10-20%)<br>◯ Severe (>20%)
Did the patient receive any transfusion of blood products?	◯ Yes<br>◯ No

© 2023 Wu AG et al. *JAMA Network Open*

Was the patient re-intubated after a planned extubation?	◯ Yes<br>◯ No
Did the patient have an unplanned extubation?	◯ Yes<br>◯ No
Did the patient travel off the unit?	◯ Yes<br>◯ No

If Yes to 'Travel off the Unit' please describe the location	☐ Radiology<br>☐ OR<br>☐ Other
Did the patient have a major surgical procedure (in the OR, unit, CATH lab, or IR)?<br>Note: Use what your hospital would define as a major surgical procedure & be consistent.	◯ Yes<br>◯ No
If Yes, please list the procedure	__________________________________

Infection related questions

Did the patient have a hospital onset infection within 2 calendar days pre/post VAE event (CLABSI, SSI, etc.)?	◯ Yes<br>◯ No
If your response is 'YES' to hospital onset infection: select the type of event(s).	☐ CLABSI<br>☐ SSI<br>☐ Viral infection<br>☐ Sepsis<br>☐ CAUTI<br>☐ Other

© 2023 Wu AG et al. *JAMA Network Open*

Was a new antibiotic started within 2 days pre/post VAE event?	◯ Yes<br>◯ No
Was the antibiotic given for 4 or more days.	◯ Yes<br>◯ No

Considerations for team discussion (IP, RT, RN, MD's)

NEED FOR MECHANICAL VENTILATION

Did the team routinely assess the appropriateness of non-invasive ventilation vs. invasive mechanical ventilation?	◯ Yes<br>◯ No
Was there active discussion on each shift to evaluate extubation readiness?	◯ Yes<br>◯ No
Does your team believe that this patient could have been extubated earlier?	◯ Yes<br>◯ No<br>◯ Unsure

© 2023 Wu AG et al. *JAMA Network Open*

WEANING PROTOCOLS	
Was this patient on a ventilator weaning protocol?	◯ Yes<br>◯ No
Was this patient on a sedation weaning protocol?	◯ Yes<br>◯ No
Would the patient have benefited from being on a ventilator and/or a sedation weaning protocol?	◯ Yes<br>◯ No<br>◯ Unsure (differing opinions)

MISSED OPPORTUNITIES:	
Does your team think that there were missed opportunities in efforts to reduce the risk of the patient developing a VAE?	◯ Yes (If YES, please comment)<br>◯ No<br>◯ Unsure (differing opinions)
Responded YES to missed opportunities (please comment)	________________________________
Does your team think that this event was preventable?	◯ Yes (if YES, please comment)<br>◯ No<br>◯ Unsure (differing opinions)

© 2023 Wu AG et al. *JAMA Network Open*
