# Supplementary material for: Pediatric Ventilator-Associated Events Before and After a Multicenter Quality Improvement Initiative
Source: JAMA Netw Open. 2023 Dec 7;6(12):e2346545. doi: 10.1001/jamanetworkopen.2023.46545 (PMC10704274; doi:10.1001/jamanetworkopen.2023.46545)
Supplement: Supplement 2. — Nonauthor Collaborators [file jamanetwopen-e2346545-s002.pdf]

Supplemental Online Content: Nonauthor Collaborators
*First name, last name, and suffix (if applicable) are required and will appear in PubMed.

***Group Name(s): Solutions for Patient Safety (SPS) PedVAE Study Group**							
***First Name and Middle Initial(s)**	***Last Name**	***Suffix (eg, Jr, III)**	Academic Degrees	Institution	Location (city, state/province, country)	Role or Contribution, eg, chair, principal investigator	Group (if more than 1 Group listed in the byline) and/or Subgroup (eg, Steering Committee)
Kevin J.	Bullock		RRT-NPS	Boston Children's Hospital	Boston, MA, USA	SPS PedVAE Study Group Investigator	
Lori	Grassmyer		MBA, RCP, RRT_NPS	Valley Children's Hospital	Madera, CA, USA	SPS PedVAE Study Group Investigator	
Lia	Lowrie		MD	SSM Health Cardinal Glennon Children's Hospital	St Louis, MO, USA	SPS PedVAE Study Group Investigator	
Elizabeth H.	Mack		MD, MS	MUSC Children's Hospital	Charleston, SC, USA	SPS PedVAE Study Group Investigator	
Stacey Morgan	Schlafly		MPH	Monroe Carell Jr Children's Hospital at Vanderbilt	Nashville, TN, USA	SPS PedVAE Study Group Investigator	
Bonnie	Seitz		MS, RN, CNS, CPN	Upstate Golisano Children's Hospital	Syracuse, NY, USA	SPS PedVAE Study Group Investigator	
Ryan	Stecks		BS, LRCP, RRT, RRT-NPS	Arkansas Children's Hospital	Little Rock, AR, USA	SPS PedVAE Study Group Investigator	
Laurel	White		MBA, BSRT, RRT, RRT-NPS	UW Health American Family Children's Hospital	Madison, WI, USA	SPS PedVAE Study Group Investigator	
