# Supplementary material for: Pediatric Ventilator-Associated Events Before and After a Multicenter Quality Improvement Initiative
Source: JAMA Netw Open. 2023 Dec 7;6(12):e2346545. doi: 10.1001/jamanetworkopen.2023.46545 (PMC10704274; doi:10.1001/jamanetworkopen.2023.46545)
Supplement: Supplement 3. — Data Sharing Statement [file jamanetwopen-e2346545-s003.pdf]

Data Sharing Statement

Wu. Pediatric Ventilator-Associated Events Before and After a Multicenter Quality Improvement Initiative. *JAMA Netw Open*. Published December 07, 2023.
doi:10.1001/jamanetworkopen.2023.46545

Data
Data available: No
